# Supplementary figures and images for: Polo-like kinase 2 inhibition reduces serine-129 phosphorylation of physiological nuclear alpha-synuclein but not of the aggregated alpha-synuclein
Source: PLoS One. 2021 Oct 6;16(10):e0252635. doi: 10.1371/journal.pone.0252635 (PMC8494365; doi:10.1371/journal.pone.0252635)

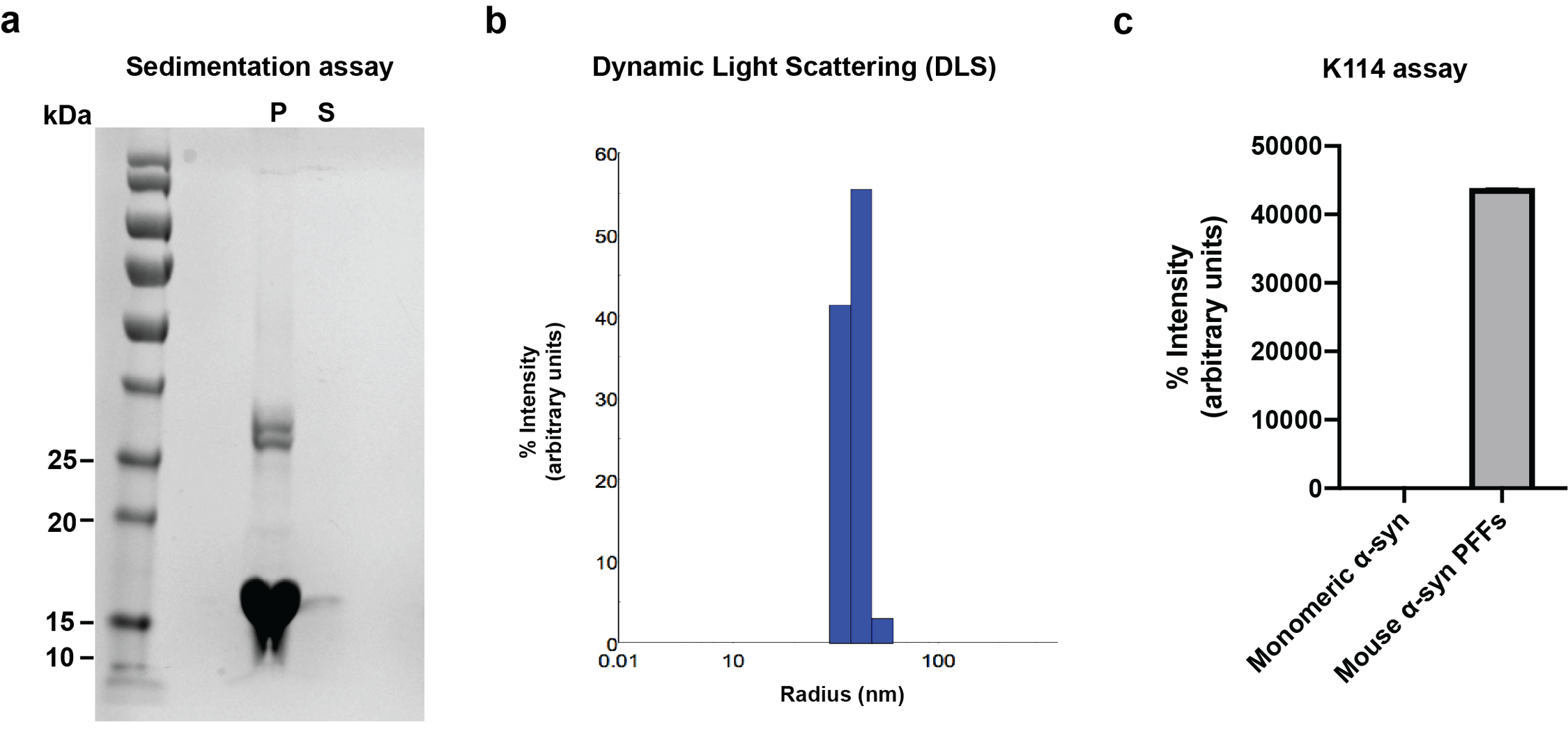

Supplement: S1 Fig — a) Biochemical characterization of mouse PFFs. The insoluble fibrils consist of pure α-syn as demonstrated by SDS-PAGE and Coomassie blue staining (P = pellet, S = supernatant). b) The sonicated mouse PFFs comprise a homogeneous, mono-dispersed particle population with a 38.8 nm radius as determined by dynamic light scattering (DLS). c) The amyloid nature of the PFFs was confirmed by a robust K114 fluorometric signal detected at 550 nm. In comparison, monomeric α-syn did not produce any signal. (TIF) [file pone.0252635.s001.tif]

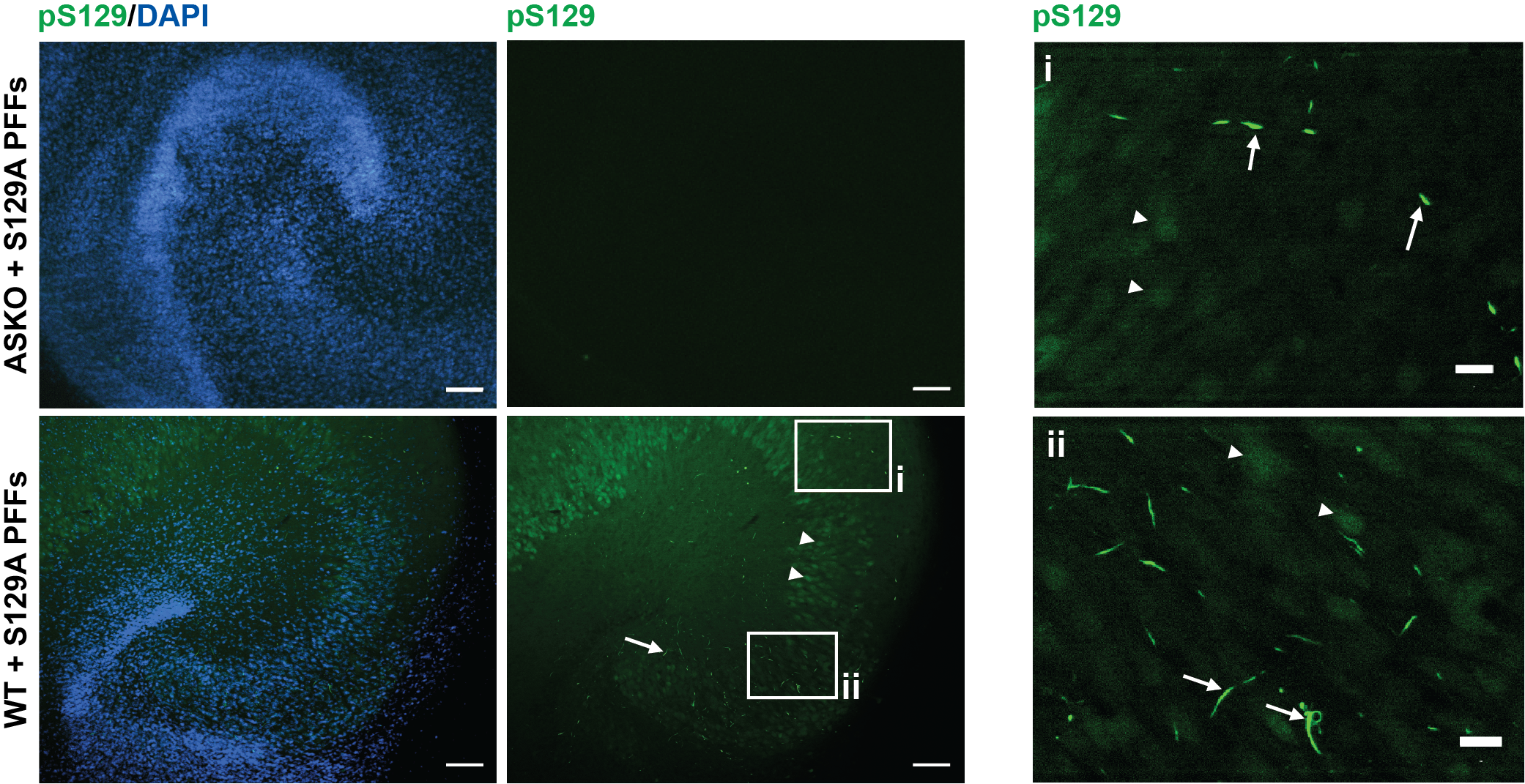

Supplement: S2 Fig — In OHSCs made from α-syn knock out (ASKO) pups and injected with S129A PFFs, immunostaining using pS129 (11A5) yields no signal at 7 dpi. In comparison, slices from WT (C57BL/6) pups injected with S129A PFFs demonstrate a nuclear pS129-signal that co-localizes with DAPI signals, predominantly in the pyramidal neurons of the CA3 and CA1 region of hippocampus (arrowheads). The nuclear signals are more diffuse and less bright than the PFF-induced axonal α-syn aggregate signals (arrows). Scale bar = 100 μm. i and ii. Magnified inserts show the bright distinct PFF-induced aggregates (arrows) and more diffuse non-aggregate-specific nuclear pS129 signal (arrowheads). Scale bars = 20 μm. (TIF) [file pone.0252635.s002.tif]

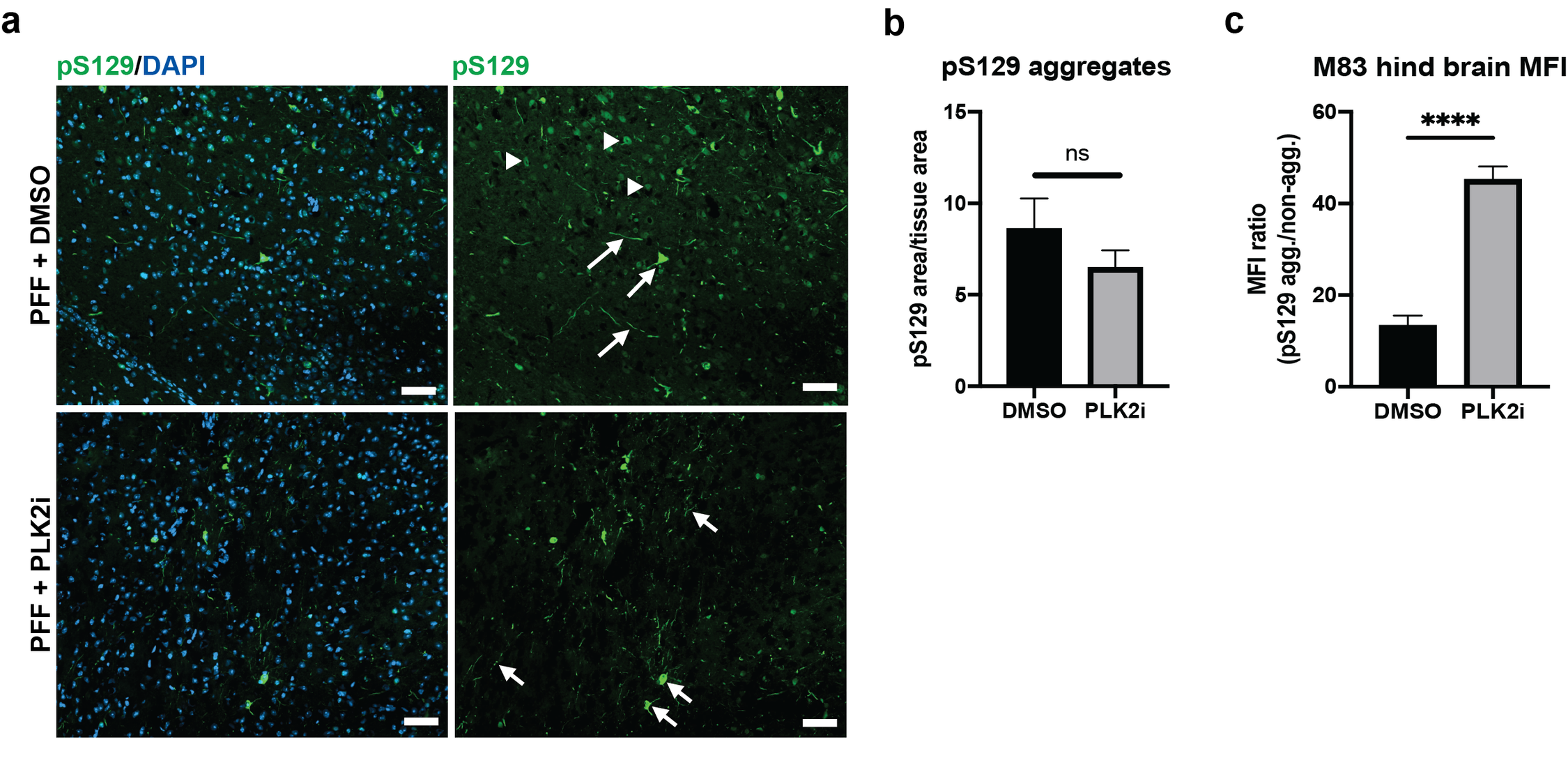

Supplement: S3 Fig — a) Representative images of α-syn aggregates detected in the hind brain of M83 mice, scale bar = 50 μm. b) Quantification of the pS129 aggregate area normalized to tissue area. Aggregate amount is unaffected by PLK2i treatment (p-value = 0.3075 using an unpaired Welch’s T test). c) PLK2i treatment facilitates easier detection of pS129-positive aggregates, as the ratio of mean fluorescence intensity of pS129 between aggregates and non-aggregate nuclear signal increases drastically upon treatment (p-value < 0.0001 by a Mann-Whitney U test). Bars represent the mean ± SD, n = 5 mice per group. (TIF) [file pone.0252635.s003.tif]

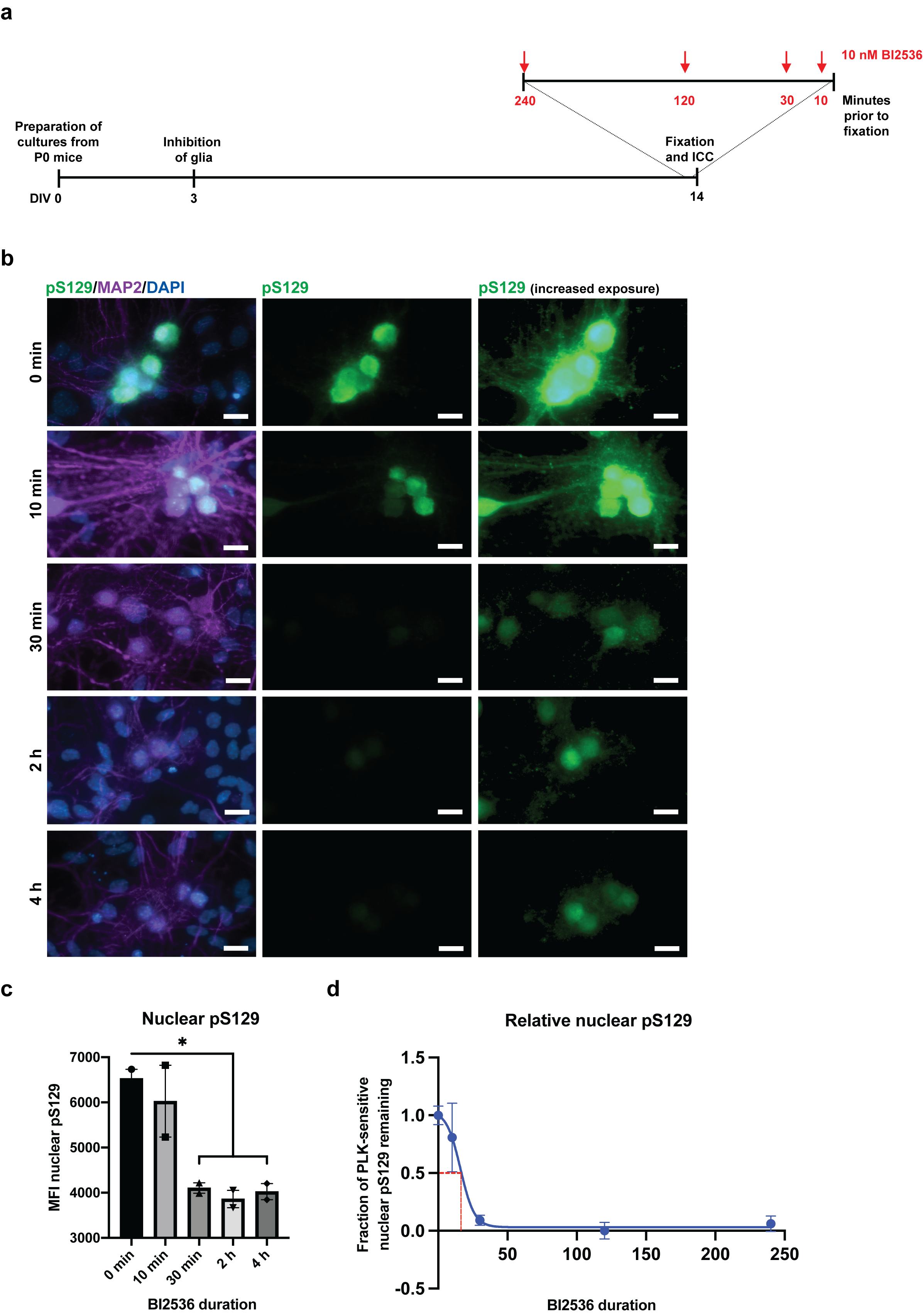

Supplement: S4 Fig — a) Experimental overview for the determination of PLK2 inhibition time course in primary hippocampal neurons. b) Representative images from hippocampal neurons cultured for 14 days and treated with 10 nM BI2536 for 0–4 hours prior to fixation. A minimum of 30 minutes treatment was sufficient to effectively decrease nuclear pS129 but not remove it completely, as is demonstrated by the increased exposure images on the right. Scale bars = 20 μm. c) Quantification of nuclear pS129-staining shows a plateauing of mean fluorescence intensity after 30 minutes of BI2536 (p-value = 0.0185). No significant decrease in nuclear pS129 was detected with 10 minutes treatment (p-value = 0.4028). Bars represent mean ± SD from 2 independent replicates and significance is indicated as * p<0.05 by one-way ANOVA followed by Holm- Šidák post-test. d) MFI values from c were normalized as fraction of PLK-sensitive nuclear pS129 relative to time point 0, allowing the determination of the approximate half-life of PLK-sensitive nuclear pS129 as 16.09 minutes (R2 for the goodness of fit = 0.8925), by fitting a 4PL sigmoidal model to the data. (TIF) [file pone.0252635.s004.tif]

**Fig.1 b**

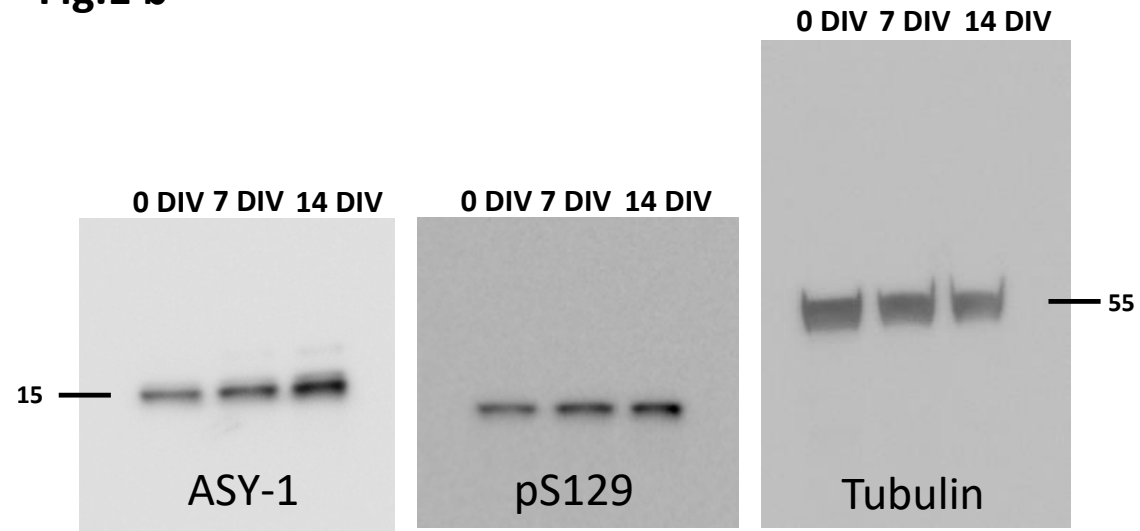

**Fig.1 c**

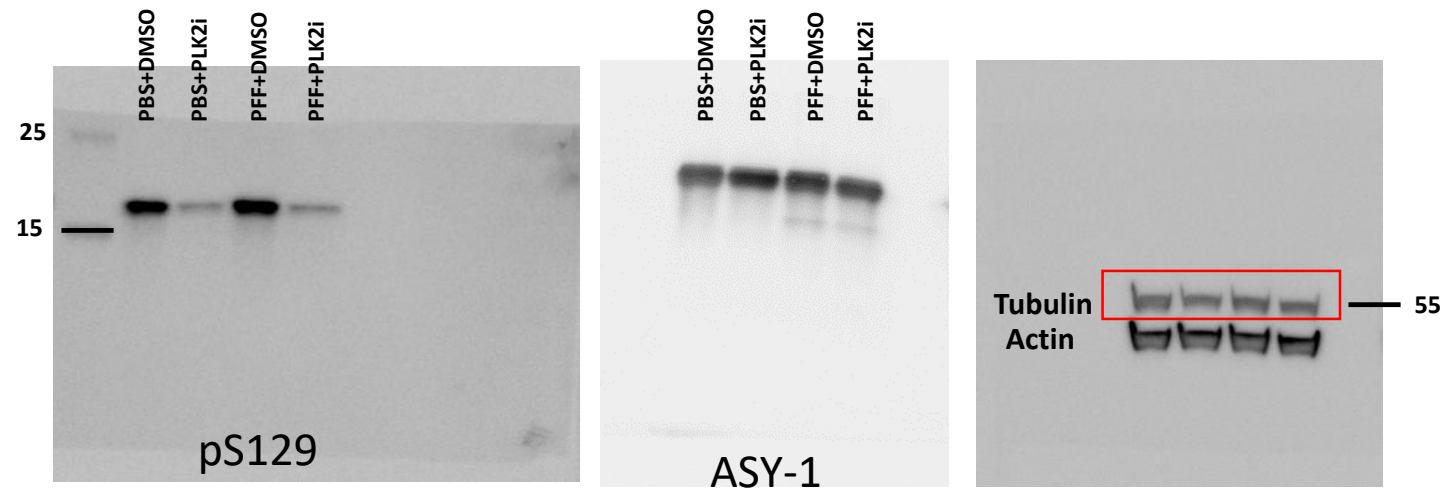

**Fig.1 f**

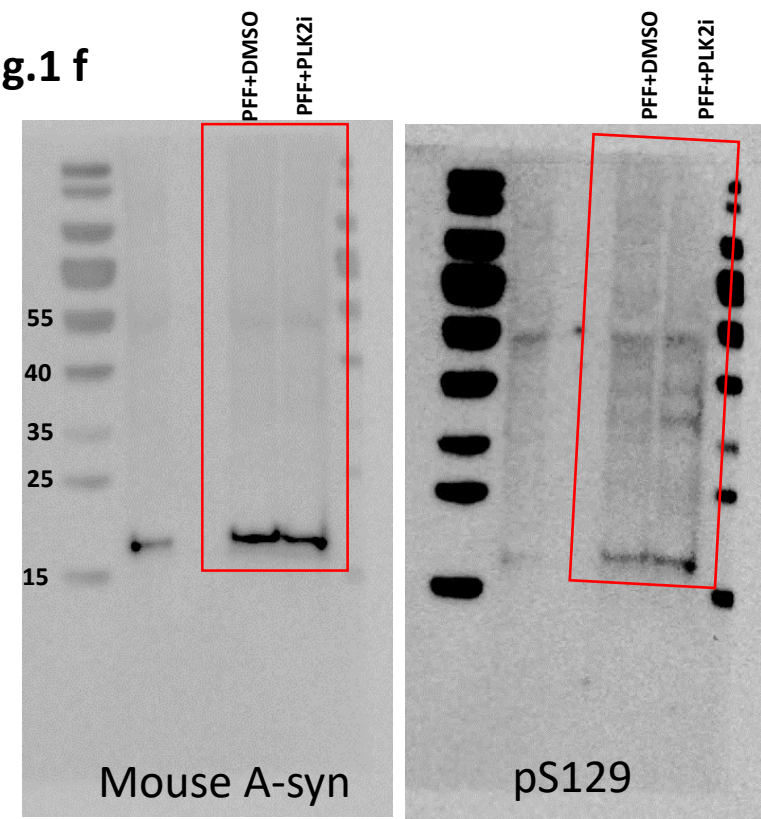

Supplement: S1 Raw images — (PDF) [file pone.0252635.s005.pdf]
